# Evaluation of an OSA Risk Screening Smartphone App in a General, Non-Symptomatic Population Sample (ESOSA)

**DOI:** 10.3390/jcm13164664

**Published:** 2024-08-08

**Authors:** J. Ulrich Sommer, Lisa Lindner, David T. Kent, Clemens Heiser

**Affiliations:** 1 Klinikum Rechts der Isar, Technical University Munich, 81675 Munich, Germany; hno@clemens-heiser.com; 2 HNO-Zentrum Mangfall-Inn, 83043 Bad Aibling, Germany; li.lindner05@gmail.com; 3 Vanderbilt University Medical Center, Nashville, TN 37232, USA; david.kent@vumc.org

**Keywords:** obstructive sleep apnea, smartphone, screening, telemedicine

## Abstract

**Background**: Obstructive Sleep apnea (OSA) is a prevalent sleep disorder, risk factor for cardiovascular disease and imposes a substantial global socioeconomic and health burden. OSA is insufficiently diagnosed as it often presents with unspecific or no symptoms. This study compares the effectiveness of a smartphone-based screening method to polysomnography (PSG) in a general, non-symptomatic population sample. **Methods**: Adult subjects were recruited from the general population. Subjects reporting OSA-related symptoms suggesting an increased OSA risk were excluded. Included subjects underwent Type-II PSG and a parallel breathing sound analysis using the Snorefox M smartphone app. The PSG scores were compared with the results of the Snorefox M app for its ability to detect moderate to severe OSA (AHI ≥ 15). **Results**: 150 subjects were included. All subjects completed the diagnostic night, no adverse events occurred. A valid analysis result was obtained for 142 subjects. A total of 24% of subjects had moderate to severe OSA based on the PSG results. The Snorefox M software app showed a sensitivity of 0.91 (0.76, 0.98), specificity of 0.83, PPV of 0.63 (0.48, 0.77), and NPV of 0.97 (0.91, 0.99) to detect AHI ≥ 15 compared with the reference PSG (95% CI). **Conclusions**: This study compares for the first time, the performance of an app-based OSA screening tool with PSG in a non-symptomatic population sample. Easily accessible screening tools can play a role in complementing existing diagnostic possibilities, helping to increase the diagnosis rate, with a positive effect on cardiovascular health in a relevant population share.

## 1. Introduction

Sleep apnea, a prevalent yet often overlooked sleep disorder, imposes a substantial global health burden along with an impact on the quality of life of millions of people worldwide [1]. Obstructive sleep apnea (OSA) is the most common disease among sleep related breathing disorders, occurring with varying degrees of severity. Interestingly, current evidence indicates that the prevalence of OSA in the general population is as high as 46% with up to 21% of cases requiring treatment, mirroring the consequences of rising obesity rates and an aging society [2].

It is well known that OSA plays a causal role in the emergence and development of cardiovascular diseases [3]. The causal role of OSA in the development of systemic hypertension is evident [4], with OSA being one of the most frequent causes of secondary hypertension, with a prevalence of nearly 80% among resistant hypertensive patients [5,6]. The benefit of treating OSA in these patients has been demonstrated [7].

Despite its widespread occurrence and potential health ramifications, OSA remains insufficiently diagnosed, as it may present with unspecific symptoms or even remain asymptomatic [8]. In the USA, approximately 80% of patients with moderate or severe OSA remain undiagnosed, causing significant healthcare and socioeconomic costs [9,10,11,12,13]. Importantly, OSA constitutes a notable contributor to excessive daytime sleepiness (EDS) and fatigue, which has also been recognized as a significant risk factor for motor vehicle accidents [14,15]. Nevertheless, a considerable proportion of the population remains unaware of their condition, due to a lack of awareness of OSA in the general population, a lack of awareness of OSA in primary care [16,17,18], and the inconvenient nature of traditional diagnostic methods. Further, the limited availability of sleep clinics and long waiting periods often impede timely diagnosis and intervention [19].

The urgent need for affordable and accessible methods for early Obstructive Sleep Apnea (OSA) detection has led to the creation of innovative smartphone-based screening solutions. These advances aim to make early diagnosis more feasible, using widespread technology to improve detection rates and patient outcomes [20].

By harnessing the capabilities of ubiquitously available mobile devices, the approach to screen patients for OSA with the help of a smartphone application aims to facilitate diagnostic access and to ensure a timely intervention if necessary. This may result in improved patient outcomes and quality of life as well as an optimized use of healthcare resources and a reduction in overall healthcare and socioeconomic costs [11].

Such low-threshold screening tools should primarily be targeting the general population, and their use should not be limited to subjects already pre-diagnosed with OSA or other sleep-related respiratory disorders. Especially, their primary role to counter OSA underdiagnosis rates in the general population should be to target subjects that do not present with OSA-typical symptoms.

To our knowledge, data comparing smartphone-based screening methods to the diagnostic gold standard, PSG, pertaining to its effectiveness in detecting OSA in the general population does not exist. This study seeks to address this deficiency by assessing the prevalence of OSA within a general population cohort that does not exhibit conventional OSA symptoms. Furthermore, it aims to determine the feasibility and reliability of a smartphone-only screening application for OSA in this demographic sample.

## 2. Materials and Methods

This prospective, non-interventional, single-arm, non-randomized observational study was designed and performed following the ISO Standard on Clinical Investigation of Medical Devices for Human Subjects, EN ISO 14155:2011 [https://www.iso.org/standard/45557.html, access on 14 June 2022]. Ethical approval was obtained from the Ethics Committee of the Bavarian State Medical Association.

### 2.1. Target Population, Recruitment and Pre-Screening

**Figure 1:**
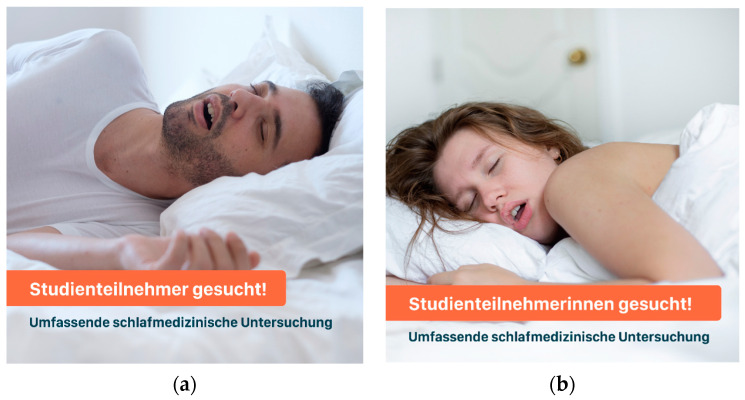
Images of the Facebook ads (**a**,**b**). English translation of the German labeling: “Study participants wanted! Comprehensive sleep medical examination”.

Volunteers were recruited though social media advertisements played to adults in the south of Germany. In addition, potential participants were targeted by email using the existing newsletter mailing list of the manufacturer. Examples of the Facebook advertisements are shown in Figure 1. In order to sample study participants which were representative of the general population, inclusion criteria were deliberately wide, with the only restrictions being age (22 or older, according to the definition of an adult by the US food and drug administration, FDA), willingness to participate, ability to use a smartphone, and typical OSA symptoms. The most pertinent symptoms associated with obstructive sleep apnea (OSA) include regular snoring, observed irregularities in breathing patterns by the bed partner, hypertension, and excessive daytime sleepiness. According to the diagnostic guidelines of the American Association of Sleep Medicine, individuals presenting with a combination of these symptoms are considered to be at an elevated risk for OSA and should be promptly referred for diagnosis to a sleep specialist [21].

Interested recipients who clicked on the ad were directed to an online pre-screening questionnaire. Pre-screening questions and inclusion criteria can be found in Table 1.

Subjects that did not meet all inclusion criteria in Section one of the questionnaire were excluded from the further recruitment process. Those meeting all inclusion criteria were asked to self-assess their OSA-related symptoms by answering Section two of the questionnaire. Subjects that answered “yes” to at least two of the questions about snoring, witnessed apnea, and hypertension, and that had an ESS score > 10, were excluded from the further enrollment process and advised to visit a healthcare professional for a diagnosis of a sleep-related breathing disorder.

The pre-screening questions in Section 2 of the questionnaire correspond to relevant symptoms that are related to OSA. Other OSA screening tools to identify at-risk subjects include the STOP–BANG, STOP, and NoSAS questionnaires. The STOP–BANG questionnaire, derived from the STOP questionnaire, includes eight components: snoring, tiredness, witnessed apneas, hypertension, body mass index (BMI), age, neck circumference, and sex. Each component is scored, with higher scores indicating a greater risk of OSA.

The STOP questionnaire focuses on four key symptoms: snoring, tiredness, witnessed apneas, and hypertension. It offers a quick assessment but with less detail than the STOP–BANG questionnaire. The NoSAS questionnaire simplifies this further including only neck circumference, obesity, snoring, age, and sex. It provides a streamlined risk assessment.

These tools are designed for easy integration into clinical practice, permitting the early identification of individuals with OSA. Both the STOP–BANG and STOP questionnaires have a high sensitivity, however, their specificity is only moderate. The NoSAS questionnaire offers a concise alternative for primary care settings.

Subjects that met all pre-screening inclusion criteria were asked to register by providing contact information, and their preferred method of contact (email or phone). All registered subjects were provided with participant information and the informed consent form for their information.

The screening process was designed to identify subjects in the general population who met the criteria for inclusion based on a simple questionnaire (see above). When assessing the general population with a screening tool, it is important to ensure that such a questionnaire is incorporated into the tool itself to avoid multiple diagnoses being clinically insignificant in these subjects.

All subjects that provided contact information were contacted using their preferred way of contact in the chronological order of registration. Questions were clarified individually by a study investigator and appointments were offered for participation in the study.

### 2.2. Observational Procedure

Participants were instructed to arrive at the site early in the evening of the day of the appointment for preparation for the observational night. All participants that showed up on the agreed date were offered the possibility to clarify any open questions with a study investigator before signing the informed consent and enrollment. Enrolled participants were then assigned a single room for the night and were interviewed by the study personnel regarding demographic information, ethnicity, general and OSA-specific medical history. Further, the pre-screening questions were repeated in order to identify subjects whose symptoms had worsened between pre-screening and the day of participation.

### 2.3. OSA Screening Smartphone App

The participants were provided with a smartphone by the investigator (iPhone XR, Apple, Cupertino, CA, USA) with the pre-installed OSA screening app Snorefox M (Diametos, Potsdam, Germany). Participants were advised to initiate a screening measurement and to place the smartphone according to the instructions given by the app, and to stop the measurement on the next morning after waking up.

**Figure 2:**
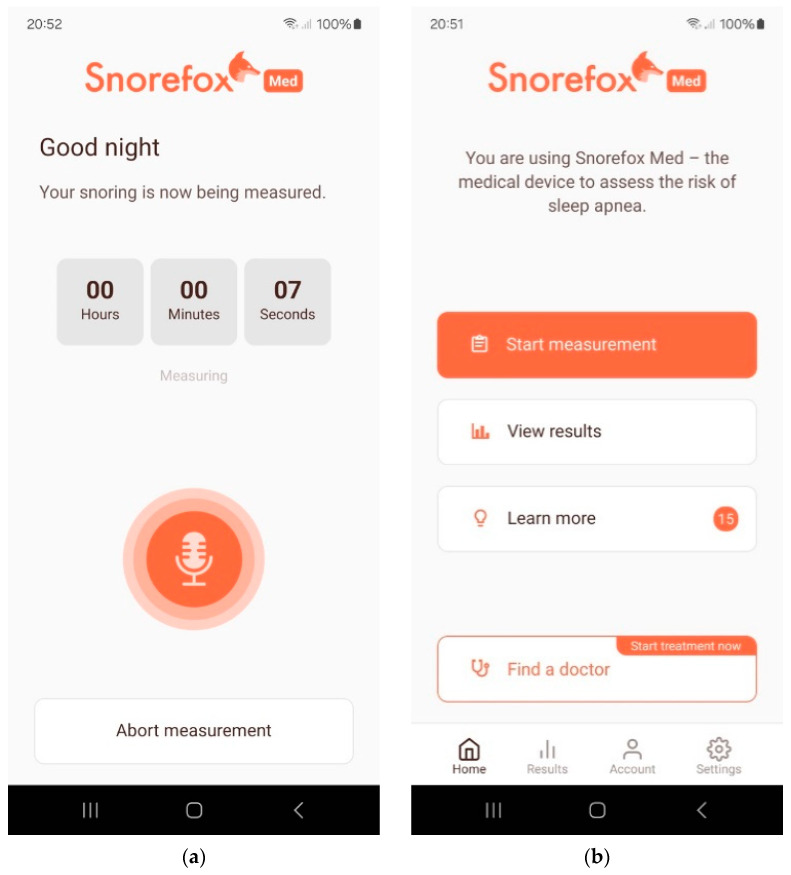
Selected smartphone screens from the Snorefox M application to illustrate the user flow (**a**) Screen after starting the measurement (**b**) Main menu.

Figure 2 shows some smartphone screens from the Snorefox M app that illustrate the user flow.

Snorefox M is mobile application software to screen for the risk of obstructive sleep apnea. Snorefox M records and registers the patterns of nocturnal breathing sounds via the smartphone microphone. The Snorefox M software [https://snorefox.com/ Access date: 14 June 2022] algorithm screens for respiratory anomalies that have occurred during the night from the acoustic patterns of the breathing sounds to form an estimate of the apnea–hypopnea index (AHI) for that user for a single night. The OSA screening risk analysis is displayed to the user as a color-coded indication of the result.

The Snorefox M software application is a CE marked device according to the European Medical Device Directive (MDD). It is intended to be used by adult medical laypersons to assess the risk of obstructive sleep apnea within the framework of a preliminary examination (screening). The software is not intended for direct diagnosis. The final diagnosis or exclusion of a sleep-related breathing disorder is made exclusively by the attending physician within the framework of a guideline-based diagnosis.

### 2.4. Reference Measurement

In parallel to the use of the Snorefox M application, a Type II polysomnography (PSG) was carried out on all participants on the same night (Sonata, Löwenstein Medical, Germany). Table 2 lists the physiological channels recorded. The reference PSG was used to record the relevant information that is required to score for the severity of an obstructive sleep apnea according to the AASM scoring criteria [22].

### 2.5. Evaluation and Follow-Up

All PSG recordings were evaluated by two certified sleep technicians manually using annotation software (MiniScreen Version 5.21a, Dr. Fenyves und Gut GmbH, Rangendingen, Germany). In case of discrepancies in the OSA severity score (i.e., AHI ≥ 15 or AHI < 15), a third sleep technician evaluated the PSG recording, and a majority vote was taken. All sleep technicians were blinded against each other and against the results of the Snorefox M app.

The scoring of the reference PSG was performed according to the AASM Manual for the Scoring of Sleep and Associated Events Version 3 [22], using an oxygen desaturation index (ODI) > 3 threshold for determination of a hypopnea. An AHI ≥ 15 was considered moderate OSA, an AHI ≥ 30 was considered severe OSA. According to [23], the first decimal place was taken into account when calculating the AHI, i.e., an AHI up to 14.9 was considered AHI < 15.

All participants were offered the possibility to make an appointment for a consultation with a sleep specialist for the evaluation of the PSG results of the observational night and a professional sleep-related breathing disorder diagnosis. If a sleep-disordered breathing disease was suspected after the observational night, the respective participants were advised by the attending healthcare professional to seek further medical treatment.

### 2.6. Study Endpoints

Primary endpoints of the study were the sensitivity and specificity of the Snorefox M software application to detect moderate to severe obstructive sleep apnea defined as an AHI ≥ 15 compared with a type II reference polysomnography performed in parallel.

Considering the intended use of the application for the screening of OSA to refer a user into a guideline-based diagnostic workup, the sensitivity and specificity to detect an AHI ≥ 15 is most important from a user safety perspective. At this background, the study was considered successful when a target sensitivity of 80% and a target specificity of 60% was reached or exceeded as co-primary endpoints.

Secondary Endpoints were positive predictive value (PPV) and negative predictive value (NPV) of the Snorefox M software application to detect an AHI ≥ 15 compared with a reference PSG.

Additionally, the following supporting analyses were made:

(a) number of participants without a study result due to not completing the study, user mistakes, or measurement failures of the PSG measuring device or the Snorefox M measurement were evaluated as a secondary endpoint.
(b) performance of the Snorefox M software application to detect an AHI ≥ 15 on the entirety of all subjects for whom a full night PSG result is provided, including those in which the Snorefox M device did not provide a result, including the latter cases in the denominator for the calculation of the sensitivity and specificity with corresponding confidence intervals.
(c) subgroup analysis for significant differences in the performance endpoints for sex, age, body mass index (BMI), and ethnicity.

### 2.7. Statistics

All statistical analyses were performed using R, version 4.3.2 (The R Foundation for Statistical Computing, Vienna, Austria). Sensitivity, specificity, positive predictive value (PPV) and negative predictive value (NPV) were calculated according to the formulas in Table 3 with 95% confidence intervals.

The primary analysis population is a modified Intent to Diagnosis population, including all subjects for whom the data points according to Table 4 are available.

Two primary effectiveness evaluations were conducted. The first primary effectiveness endpoint assessment was the sensitivity of the Snorefox M software application to detect an AHI ≥ 15 compared with a reference polysomnography performed in parallel. The second primary effectiveness endpoint assessment was the specificity of the Snorefox M software application to detect an AHI ≥ 15 compared with a reference polysomnography performed in parallel.

The PPV and NPV were calculated based on the actual prevalence in the study population.

The minimum sample size needed to show the expected sensitivity (80%) and expected specificity (60%) was calculated with a delta = 0.2, which was analyzed with a one sided 90% confidence interval, one for sensitivity and one for specificity (one-sided level of significance = 2.5%, calculation of the lower border of the 90% confidence interval) with a power of 90%. The expected prevalence in the study population of approximately 36% OSA was estimated by data that was collected in a representative user cohort of the product outside of a clinical trial.

In total, a sample size of at least 142 subjects completing the study was calculated to show the expected values to be significant. Estimating a dropout rate of 5% of participants not completing the study, due to user mistakes, or due to technical failure of the measuring devices, a total number of 150 study participants was determined.

The subgroups for the supporting subgroup analysis were created according to sex, age and BMI. Age and BMI were grouped via a median split across all enrolled subjects. Prevalence, sensitivity, specificity, PPV and NPV were then calculated for all subjects in each subgroup for which full analysis results were available and reported with 95% confidence intervals. Additionally, prevalence was compared between the two subgroups via Fisher’s exact test, respectively. A level of significance of 5% was applied.

## 3. Results

### 3.1. Pre-Screening

A total of 611 responses were collected using the online pre-screening and self-assessment questionnaire. A total of ten respondents (1.6%) were excluded because they did not meet all inclusion criteria. Of the remaining 601 respondents, 140 (23.0%) were excluded in the symptom self-assessment step because they reported an ESS score greater than 10 and two of the symptoms if snoring, witnessed apnea, and hypertension. In total, 461 subjects were deemed eligible for the study after the pre-screening self-assessment. All eligible subjects that provided contact information were contacted in the chronological order of registration and were offered dates for participation. The first 150 subjects that accepted an offered date and that showed up at the site at the agreed date were enrolled in the study. Table 4 shows the baseline demographics of the enrolled subjects as reported on the evening of the observational night. Table 5 shows the responses to the self-assessment symptom questions on the evening of the observational night. No participant had to be excluded at this stage due to self-reported OSA-related symptoms.

### 3.2. Observational Night

All of the 150 enrolled subjects completed the observational night. No adverse events were reported. The Snorefox M did not provide a valid analysis result in three cases (2.0%). In five cases, the PSG recording could not be evaluated for AHI due to insufficient SpO_2_ sensor data (3 cases, 2.0%), and because of insufficient sleep time according to the EEG sensor data (2 cases, 1.3%). See Table 6 for a summary.

The two blinded scorers were in agreement in 143 cases regarding the AHI threshold being ≥15 or <15. In the cases of disagreement, a third scoring result by a third scorer was decisive for the majority result.

Of the 145 subjects for whom a valid PSG scoring result was available, 35 PSGs received a majority score of AHI ≥ 15, which corresponds to a prevalence of moderate or severe OSA in the study population of (24%).

**Figure 3:**
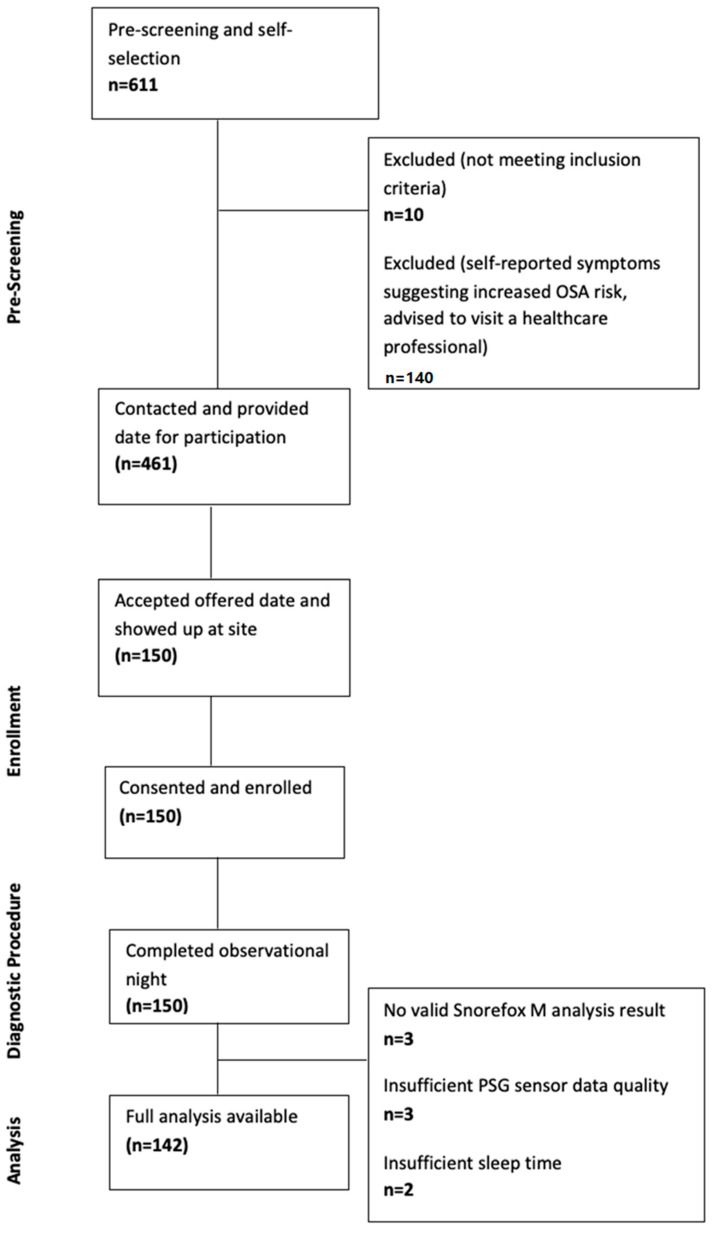
Consort diagram showing the flow of participants.

Figure 3 shows a consort diagram showing the flow of participants.

### 3.3. Performance of the Snorefox M Compared to the Reference PSG

The Snorefox M software correctly detected an AHI ≥ 15 in 31 cases, and it correctly detected an AHI < 15 in 90 cases. Considering all subjects for whom a valid Snorefox M analysis and a valid PSG result was available, Snorefox M detects moderate to severe OSA with a sensitivity of 91% and a specificity of 83% compared to a Type II polysomnography. Based on the true prevalence of 24% in the study population, this corresponds with a PPV of 63% and an NPV of 97%. A summary of the performance data including the confidence intervals can be found in Table 7.

Table 8 contains the detailed results for subgroup analyses according to sex, age and BMI. Ethnicity could not be considered as only four participants (2.7%) stated a non-white ethnicity. Age and BMI were grouped via a median split across all enrolled subjects with the cutoff 49 for age and 26.7 for BMI.

No significant differences in product performance regarding sensitivity, specificity, PPV and NPV could be found between the subgroups for sex, age, or BMI. It is of note that there are significant differences in prevalence among the subgroups, with a higher prevalence of moderate to severe OSA in men, in the older half of participants, and in the half with a higher BMI. This is not surprising and is to be expected, as generally OSA prevalence is higher in men, and increases with age and BMI [24].

## 4. Discussion

Obstructive Sleep Apnea is a highly prevalent yet severely underdiagnosed disease with an enormous socioeconomic impact.

Smartphone apps are a viable means of increasing awareness of OSA in large parts of the risk groups.

Compared with Type 3 home sleep test devices and single channel oximetry monitors, an application only requiring a smartphone offers the possibility of even wider adoption, as a Smartphone is available in nearly every household, and using an app is independent of attaching sensors or providing proprietary sensor hardware, offering logistical and cost advantages.

The value of a low-threshold screening tool lies in the availability of the tool independent of a visit to a healthcare professional. To take account for this, this study was designed to sample study participants from the general population.

The recruitment deliberately also addressed asymptomatic subjects, as a significant proportion of individuals with OSA do not exhibit typical symptoms. However, they still face significant health risks. Undiagnosed OSA can lead to serious complications such as cardiovascular diseases, hypertension, stroke, and diabetes, even in the absence of overt symptoms. Early detection can prevent these adverse outcomes by enabling timely intervention and management. To the authors’ knowledge, this is the first study to screen for the prevalence of moderate to severe OSA explicitly in an asymptomatic, general population. After excluding subjects with a combination of telltale symptoms from enrollment, an impressive 24% of asymptomatic subjects showed an AHI ≥ 15 in the observational night. The magnitude is in line with other population-based studies, showing a prevalence of 21% in the general population [2], or 35% (23% in women and 50% in men) in an adult population sample [18].

This underpins the need to provide easy-to-access means for the general population to screen for OSA even in the absence of typical symptoms that would be the trigger for a home sleep apnea test carried out by a sleep specialist.

The Snorefox M app showed, in this study, a sensitivity of 91% and a specificity of 83% to detect moderate to severe OSA, defined as AHI ≥ 15, compared with a type 2 reference polysomnography, making it a valuable tool in the portfolio of OSA screening means which are available to the sleep medical professional and to the general population.

Symptom questionnaires alone usually present a compromise between sensitivity and specificity. Other available smartphone apps offer recording and playback of nightly snoring noises. Although snoring may be a lead symptom for OSA, attempts to validate snoring recording software against PSG showed that there is a strong correlation to detect snoring duration and severity, but no statistically significant correlation with OSA severity [25,26]. Numerous wearable devices are available in the form of watches, rings, and patches. These products have in common that they are, to the most part, not validated against medical diagnostic standards for sleep disordered breathing and therefore their usefulness as part of the diagnostic journey for OSA patients cannot be seriously assessed.

The study deliberately did not exclude subjects with a previous diagnosis of a sleep-related breathing disorder. A total of 21 subjects (14%) reported a previous OSA diagnosis, of which only five (3%) were currently undergoing treatment. It is of note that eight of those prediagnosed subjects had an AHI < 15 according to the PSG performed in the diagnostic night. This means that a previous OSA diagnosis was not confirmed in 38% of those subjects. For the other 13 subjects, the OSA diagnosis could be confirmed. This is in line with previous studies reporting a high night-to-night variability of respiratory events in OSA, with as much as 49% of subjects changing OSA severity class at least once in sequential sleep studies [27]. This variability can impact the accurate diagnosis and classification of sleep apnea severity since a single night’s study might not capture the typical severity of an individual’s condition. Factors such as sleep position, alcohol consumption, and sleep stages contribute to these fluctuations. This variability underscores the importance of considering multiple nights of data for diagnosis and treatment assessment, highlighting the dynamic nature of sleep apnea and the need for personalized management strategies. A smartphone app that can easily and cost-efficiently be used for multiple nights or over a longer period of time might provide valuable information in addition to a single night diagnostic procedure.

A limitation of the study design could be a bias in the recruited population, as people who signed up for the pre-screening process are likely to have an inherent interest in their sleep health. This could be particularly the case for participants recruited from the existing newsletter mailing list. On the other hand, such a bias is inherent with most studies recruiting from the general population, as interest in participating in a trial in the first place is a fundamental prerequisite for the recruitment of participants.

Subjects with an earlier OSA diagnosis could have changed their lifestyle, so that the OSA condition might actually have improved over time and could not be confirmed. Participants were not asked about possible lifestyle changes. Experience from the clinical routine shows, however, that OSA conditions normally do not improve with age and that lifestyle changes, such as weight loss and increased physical exercise as a consequence of an OSA diagnosis are rather rare.

## 5. Conclusions

This study compares for the first time the performance of an app-based OSA screening tool in comparison with a full type II polysomnography in a sample of the general population without typical OSA symptoms.

Tools such as the Snorefox M app can play a relevant role in the diagnostic journey, as it complements the existing diagnostic possibilities with a screening option for the general population, addressing those that have an interest in their sleep health, but have not yet taken the decision to visit a doctor.

It may contribute to an earlier diagnosis of OSA, preventing the onset of symptoms or the development of comorbidities such as cardiac diseases, allowing a timely causal treatment and improving health in a relevant share of the population.

By flexibly and easily identifying the individual OSA risk, it can promote awareness of possible health risks and support the decision to visit an HCP, with a positive effect on individual health as well as positive socioeconomic effects by reducing cardiovascular sequelae as a consequence of untreated OSA.

## Figures and Tables

**Table 1:** Pre-screening questions. The questions were presented to the subjects in the German language.

Section 1 Pre-Screening Questions to Assess Inclusion Criteria	
Participants answering “no” to one or more of the questions in Section 1 were excluded from the further recruitment process.	Are you 22 years of age or older?
	2. Are you able to use a smartphone?
	3. Are you willing and interested in participating in this study? 4. Are you willing and able to sleep alone (without a bed partner) for one night? 5. Are you willing and able to sleep one night with a cell phone next to you which is turned on? 6. Are you willing and able to sleep one night while polysomnography (PSG) is being performed?
**Section 2** **Pre-Screening Questions to Self-Assess OSA-Related Symptoms**	
Pre-screening questions to self-assess OSA-related symptoms	Do you snore loudly and regularly (every night or almost every night)?
	2. Has anyone ever noticed that you stop breathing or gasp for air in your sleep? 3. Do you have high blood pressure or are you being treated for it?
In the following situations, how likely are you doze off or fall asleep, in contrast to just feeling tired? Use the following scale to choose the most appropriate number for each situation: 0 would never doze or sleep 1 slight chance of dozing or sleeping 2 moderate chance of dozing or sleeping 3 high chance of dozing or sleeping	Sitting and reading Watching TV Sitting inactive in a public place Being a passenger in a car for an hour Lying down in the afternoon Sitting and talking to someone Sitting quietly after lunch (no alcohol) Stopping for a few minutes in traffic while driving

**Table 2:** Physiological channels recorded during the reference PSG.

Physiological Channel
6 × EEG (F3/M2, F4/M1, C3/M2, C4/M1, O1/M2, O2/M1) EOG (left/right) EMG Chin (3×) ECG Leg movements (left/right) Respiration (flow, thermistor) Effort (thorax, abdomen) Oxygen saturation SpO_2_ Heart rate Pulse wave Snoring (contact microphone) Body position

**Table 3:** Formulas used for the calculation of sensitivity, specificity, PPV and NPV.

		Diagnosis According to Current Standard (Type II PSG)	
Snorefox M analysis	**Positive (OSA)**	**Positive (OSA)**	**Negative (no OSA)**
		A	B
	**Negative** **(no OSA)**	C	D
**Effectiveness Measures**		**Definition**	
Sensitivity		$\frac{A}{A+C}$	
Specificity		$\frac{D}{D+B}$	
Positive Predictive Value (PPV)		$\frac{A}{A+B}$	
Negative Predictive Value (NPV)		$\frac{D}{C+D}$	
False Negative Rate		1-sensitivity	
False Positive Rate		1-specificity	

**Table 4:** Baseline demographics of the enrolled subjects.

	Mean	Range
Age (years)	46.5	22–75
Height (cm)	172.7	149–195
Weight (kg)	83.2	45–150
BMI (kg/m^2^)	27.9	18.4–53.1
**sex**	**number of subjects**	**percent (%)**
female	83	55
male	67	45
**of those female**	**number of subjects**	**percent (%)**
pre-menopausal	48	58
post-menopausal	27	32
would not say	8	10
**ethnicity**	**number of subjects**	**percent (%)**
Hispanic	0	0.0
American Indian	0	0.0
Asian	3	2.0
Black	1	0.7
Native Hawaiian	0	0.0
White	146	97.3

**Table 5:** Responses to the symptom questionnaire on the evening of enrollment.

Question	Yes	No	% Yes
witnessed apnea	46	104	31
snoring	129	21	86
hypertension	25	125	17
ESS score	**mean** 4.3	**range** 0–10	

**Table 6:** Number of participants that did not have a study result.

	Number of Subjects
Subjects enrolled	150
Subjects that did not complete the study	0
Snorefox M did not provide a result	3
PSG invalid result (no SpO_2_ data)	3
Insufficient sleep time according to EEG	2

**Table 7:** Performance of the Snorefox M software application to detect an AHI ≥ 15 compared with a reference polysomnography performed in parallel (95% confidence intervals).

All Subjects with a Completed Snorefox M Analysis and a PSG Result (n = 142)	(95% CI)
True prevalence	0.24 (0.17, 0.32)
Sensitivity ^1^	0.91 (0.76, 0.98)
Specificity ^1^	0.83 (0.75, 0.90)
Positive predictive value	0.63 (0.48, 0.77)
Negative predictive value	0.97 (0.91, 0.99)

^1^ primary study endpoint.

**Table 8:** Subgroup analyses (95% confidence intervals) for all subjects with a completed Snorefox M analysis and a PSG result (n = 142).

Subgroup: Sex	Male	Female	
Total subjects (n)	64/142 (45%)	78/142(55%)	*p* = 0.003
True prevalence	0.36 (0.24, 0.49)	0.14 (0.07, 0.24)	
Sensitivity	0.91 (0.72, 0.99)	0.91 (0.59, 1.00)	
Specificity	0.80 (0.65, 0.91)	0.85 (0.74, 0.93)	
PPV	0.72 (0.53, 0.87)	0.50 (0.27, 0.73)	
NPV	0.94 (0.81, 0.99)	0.98 (0.91, 1.00)	
**Subgroup: age (split at median)**	**lower half**	**upper half**	
Mean (range)	34.9 (22–48)	57.6 (49–75)	*p* < 0.001
Total subjects	71/142	71/142	
True prevalence	0.08 (0.03, 0.17)	0.39 (0.28, 0.52)	
Sensitivity	1.00 (0.54, 1.00)	0.89 (0.72, 0.98)	
Specificity	0.88 (0.77, 0.95)	0.77 (0.61, 0.88)	
PPV	0.43 (0.18, 0.71)	0.71 (0.54, 0.85)	
NPV	1.00 (0.94, 1.00)	0.92 (0.78, 0.98)	
**Subgroup: BMI (split at median)**	**lower half**	**upper half**	
Mean (range)	23.3 (18.4–26.3)	32.1 (26.4–53.1)	*p* < 0.001
Total subjects (n)	71/142	71/142	
True prevalence	0.10 (0.04, 0.19)	0.38 (0.27, 0.50)	
Sensitivity	0.86 (0.42, 1.00)	0.93 (0.76, 0.99)	
Specificity	0.84 (0.73, 0.92)	0.82 (0.67, 0.92)	
PPV	0.38 (0.15, 0.65)	0.76 (0.58, 0.89)	
NPV	0.98 (0.90, 1.00)	0.95 (0.82, 0.99)	

No significant difference in product performance (sensitivity, specificity, PPV and NPV) could be found between the subgroups (*p* > 0.05 for all subgroups).

## Data Availability

The original contributions presented in the study are included in the article, further inquiries can be directed to the corresponding author.
